# Adverse Effects, Transformation and Channeling of Aflatoxins Into Food Raw Materials in Livestock

**DOI:** 10.3389/fmicb.2019.02861

**Published:** 2019-12-11

**Authors:** Ferenc Peles, Péter Sipos, Zoltán Győri, Walter P. Pfliegler, Federica Giacometti, Andrea Serraino, Giampiero Pagliuca, Teresa Gazzotti, István Pócsi

**Affiliations:** ^1^Institute of Food Science, Faculty of Agricultural and Food Sciences and Environmental Management, University of Debrecen, Debrecen, Hungary; ^2^Institute of Nutrition, University of Debrecen, Debrecen, Hungary; ^3^Department of Molecular Biotechnology and Microbiology, Institute of Biotechnology, Faculty of Science and Technology, University of Debrecen, Debrecen, Hungary; ^4^Department of Veterinary Medical Sciences, University of Bologna, Bologna, Italy

**Keywords:** aflatoxin, *Aspergillus*, storage conditions, mitigation strategies, livestock

## Abstract

Aflatoxins are wide-spread harmful carcinogenic secondary metabolites produced by *Aspergillus* species, which cause serious feed and food contaminations and affect farm animals deleteriously with acute or chronic manifestations of mycotoxicoses. On farm, both pre-harvest and post-harvest strategies are applied to minimize the risk of aflatoxin contaminations in feeds. The great economic losses attributable to mycotoxin contaminations have initiated a plethora of research projects to develop new, effective technologies to prevent the highly toxic effects of these secondary metabolites on domestic animals and also to block the carry-over of these mycotoxins to humans through the food chain. Among other areas, this review summarizes the latest findings on the effects of silage production technologies and silage microbiota on aflatoxins, and it also discusses the current applications of probiotic organisms and microbial products in feeding technologies. After ingesting contaminated foodstuffs, aflatoxins are metabolized and biotransformed differently in various animals depending on their inherent and acquired physiological properties. These mycotoxins may cause primary aflatoxicoses with versatile, species-specific adverse effects, which are also dependent on the susceptibility of individual animals within a species, and will be a function of the dose and duration of aflatoxin exposures. The transfer of these undesired compounds from contaminated feed into food of animal origin and the aflatoxin residues present in foods become an additional risk to human health, leading to secondary aflatoxicoses. Considering the biological transformation of aflatoxins in livestock, this review summarizes (i) the metabolism of aflatoxins in different animal species, (ii) the deleterious effects of the mycotoxins and their derivatives on the animals, and (iii) the major risks to animal health in terms of the symptoms and consequences of acute or chronic aflatoxicoses, animal welfare and productivity. Furthermore, we traced the transformation and channeling of Aspergillus-derived mycotoxins into food raw materials, particularly in the case of aflatoxin contaminated milk, which represents the major route of human exposure among animal-derived foods. The early and reliable detection of aflatoxins in feed, forage and primary commodities is an increasingly important issue and, therefore, the newly developed, easy-to-use qualitative and quantitative aflatoxin analytical methods are also summarized in the review.

## Introduction

Mycotoxins are harmful secondary metabolites produced by a variety of mold species that represent serious health risks to both humans and household animals (Beardall and Miller, 1994) and, not surprisingly, they cause both acute and chronic diseases called mycotoxicoses. The chronic pathological conditions develop over a longer period of time through the consumption of both cereals and animal products, e.g., milk, meat, and eggs. They represent a risk factor to human health directly in the food chain and through biological transformations as well. Mycotoxinogenic fungi are present mainly in small grains like wheat, barley, rye, rice, triticale, and corn (Miller, 2008; Gacem and El Hadj-Khelil, 2016; Udovicki et al., 2018) and also in different feedstuffs. In fact, aflatoxins were first discovered following a severe livestock poisoning incident in England involving turkeys (e.g., Amare and Keller, 2014; Keller, 2019). In addition, aflatoxins may also occur in peanuts, figs, pistachios, Brazil nuts and cottonseeds.

**FIGURE 1:**
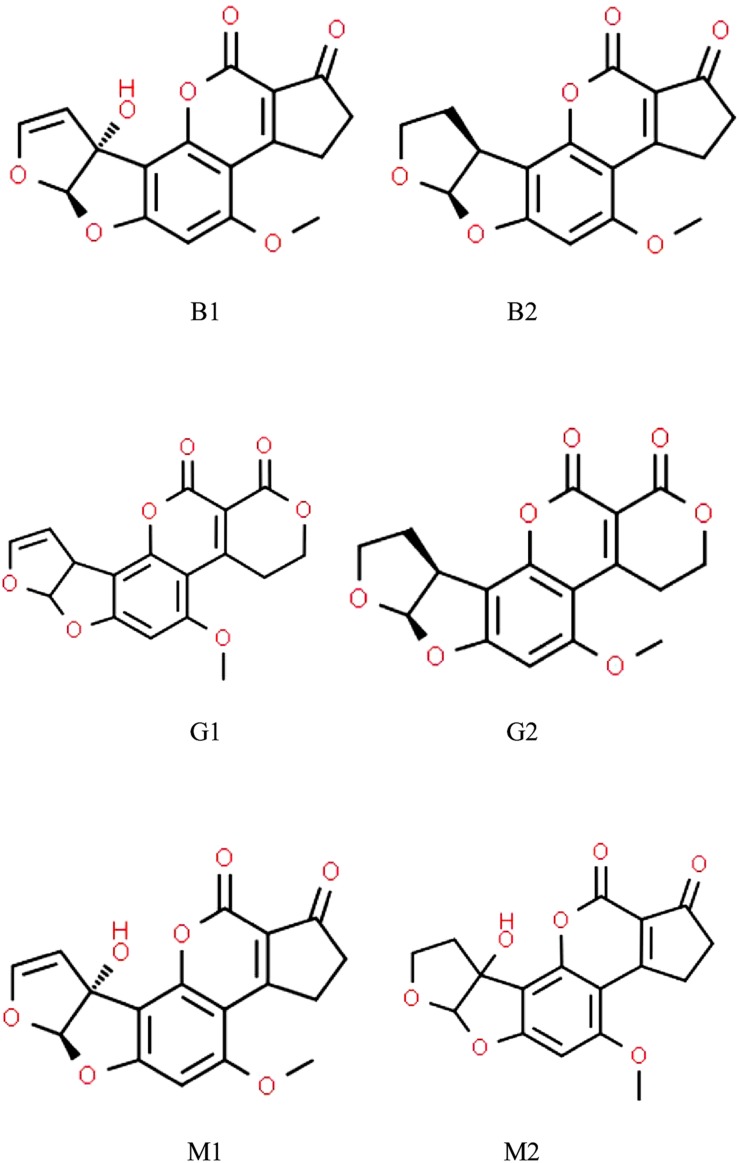
Chemical structures of aflatoxins most frequently found in animal husbandry.

A number of *Aspergillus* spp. belonging to sections *Flavi*, *Ochraceorosei* and *Nidulantes* have the ability to produce the harmful, carcinogenic difuranocoumarin derivatives called aflatoxins (Varga et al., 2015; Chen A.J. et al., 2016; Niessen et al., 2018; Frisvad et al., 2019). *Aspergillus flavus*, *Aspergillus parasiticus*, and *Aspergillus nominus* are the most often detected aflatoxigenic Aspergilli in feed (Table 1). Aflatoxin producer Aspergilli are of paramount importance because the aflatoxins synthesized by them are among the strongest naturally occurring carcinogenic substances (Kumar et al., 2008). Considering their chemical structures, aflatoxins are furanocoumarin derivatives (Figure 1), of which aflatoxin M_1_ (AFM_1_), a hydroxylated derivative of aflatoxin B_1_ (AFB_1_), occurs in milk and in various dairy products (Prandini et al., 2009; Giovati et al., 2015). AFM_1_ is a distinguished target in on-going mycotoxin-related research, because AFM_1_ consumption may be exceptionally dangerous for children especially at younger ages (Udomkun et al., 2017; Rodríguez-Blanco et al., 2019; Ojuri et al., 2019).

**TABLE 1:** Aflatoxin producer *Aspergillus* species detected in feed.

**Country**	**Type of feed**	**Isolated *Aspergillus* spp.**	**References**
Argentina	Maize silage, corn grains, cotton seed, finished feed	*A. flavus, A. parasiticus*	Alonso et al., 2009
Argentina	Maize silage	*A. flavus, A. parasiticus*	González Pereyra et al., 2011
Brazil	Concentrated feed and maize silage	*A. parasiticus*, *A. nomius*	Variane et al., 2018
Egypt	Maize silage	*A. flavus*	El-Shanawany et al., 2005
France	Maize silage	*A. parasiticus*	Garon et al., 2006
Ghana	Corn grain	*A. flavus*	Dadzie et al., 2019
Indonesia	Maize of livestock feed	*A. flavus*	Sukmawati et al., 2018
Iran	Silage, concentrate, hay, TMR	*A. flavus*	Davari et al., 2015
Malaysia	Corn grains	*A. flavus*	Zulkifli and Zakaria, 2017
Malaysia	Wheat and barley	*A. flavus*	Reddy and Salleh, 2010
Pakistan	Feed samples	*A. flavus, A. parasiticus*	Usman et al., 2019
Saudi Arabia	Animal feedstuff samples	*A. flavus, A. parasiticus, A. nomius*	Gherbawy et al., 2019
Serbia	Corn, wheat, barley, soybean and sunflower grains	*A. flavus*	Lević et al., 2013
Spain	Barley grains	*A. flavus, A. parasiticus*	Mateo et al., 2011
Tanzania	Corn grains	*A. flavus*	Manoza et al., 2017

The risks associated with mycotoxins have an enormous economic impact, which heavily supports the need for further research in this field (Gnonlonfin et al., 2013). The scope of future mycotoxin-linked studies should be broadened and should focus more on the prevention of mycotoxin production and the reduction of their deleterious effects. One of the major objectives of current investigations is the breeding and cultivation of novel plant varieties/hybrids more resistant to infections by mycotoxin producer fungi. Another major goal focuses on the accuracy of the storage of crops and crop products, especially silage, to control the production of mycotoxins more tightly (Driehuis et al., 2018; Ogunade et al., 2018; Glamočić et al., 2019). A further important step in mycotoxin control would be to make feeding practices more rigorous to prevent mycotoxins from entering the body of animals in the first place (Aslam et al., 2016; Shanakhat et al., 2018). Furthermore, countermeasures may also include the application of various mycotoxin binding agents mixed with the feed (De Mil et al., 2015; Vila-Donat et al., 2018). Besides agricultural and technological approaches combating aflatoxins successfully, we also need to develop more sensitive and more reliable analytical methods (Kos et al., 2016).

To eradicate or at least to decrease mycotoxins considerably in the feed and food chain is undoubtedly a high-complexity and highly prestigious aim, which absolutely requires the effective cooperation of experts working in different fields. Such expanding co-operations will hopefully help on-going research obey the “from farm to fork” principle more. In this case, this concept means that we need to deal not only with production, storage and processing issues but also their impacts on human health as well (Fink-Gremmels, 2008b; Ogunade et al., 2014; Asemoloye et al., 2017).

In this review, we focus on special parts of the feed and food chain like silage production and mitigation of mycotoxins by microbial products. A special attention will be paid to novel findings, which may help the feed management in animal husbandry to prevent and alleviate aflatoxin contamination. Other major issues tackled by this review include new pieces of information on the deleterious physiological effects of aflatoxins on domestic animals, which help us further in proper risk assessment and management. Moreover, up-to-date analytical tools and methods to measure aflatoxins precisely both on farms and analytical laboratories will also be covered. We hope that shedding light on the high-complexity relations between aflatoxin producer Aspergilli, aflatoxin contaminations in feeds and feeding practices in animal husbandry will also give us new hints on the efficient control of aflatoxin contaminations in feeds and minimizing the carry-over of these harmful myctotoxins to humans through the food chain.

## Aflatoxin Production in Fungi: Biosynthesis and Regulation

Considering the aflatoxin biosynthetic pathway acetate molecules are converted to norsoloinic acid at first by two fatty acid synthases, a polyketide synthase and a monooxygenase (Ehrlich et al., 2010; Yu, 2012; Roze et al., 2013). The biosynthesis proceeds through the intermediates averantin, averufin, versiconal and branches at versicolorin B to give rise to aflatoxin B_1_ and G1 *via* the versicolorin A/sterigmatocystin and to aflatoxin B_2_ and G_2_ *via* the versicolorin B/dehydrosterigmatocystin pathways, respectively (Yu, 2012). The letters B and G stand for the blue and green fluorescence of these compounds observable under ultraviolet light, when separated by thin-layer chromatography (Yu, 2012). The aflatoxin biosynthetic gene cluster is sophisticatedly regulated by both local (AflR and AflS) and global (Velvet Complex) regulatory elements (Amaike and Keller, 2011; Alkhayyat and Yu, 2014; Amare and Keller, 2014; Gil-Serna et al., 2019; Keller, 2019). Environmental factors like the availability of carbon and nitrogen sources, changing pH, temperature and light conditions as well as variations in the redox status of the fungal cells all have their impacts on aflatoxin production (Alkhayyat and Yu, 2014). Among environmental stresses, oxidative stress seems to play a pivotal role in the initiation of aflatoxin production (Reverberi et al., 2010; Hong et al., 2013; Roze et al., 2013; Amare and Keller, 2014). Plant–fungus interactions also affect the biosynthesis of aflatoxins e.g., through oxylipin production, which have been reviewed e.g., by Pusztahelyi et al. (2015). Undoubtedly, a deeper understanding of the elements and regulation of the aflatoxin biosynthetic gene clusters operating in aflatoxigenic fungi is an important prerequisite for the development of novel and successful mycotoxin control strategies in the future (Alkhayyat and Yu, 2014; Gil-Serna et al., 2019).

## Fungal Activity and Aflatoxin Production in Stored Grains

**FIGURE 2:**
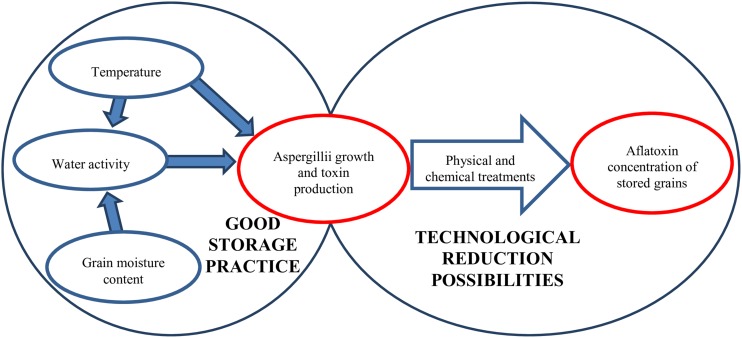
Factors influencing the aflatoxin content of grains during storage.

Aflatoxin-producing Aspergilli (Varga et al., 2015; Chen A.J. et al., 2016; Niessen et al., 2018; Frisvad et al., 2019) may originate from crop fields but post-harvest infections have also been reported (Gachara et al., 2018). Aflatoxin production cannot be linked strictly to any specific phase of growth or processing status although poorly managed post-harvest conditions during drying and storage can result in rapid increase in mycotoxin concentrations (Hell et al., 2010; Chulze, 2010). Grain drying is costly but selecting a variety or hybrid optimal for a given crop field can help farmers to harvest cereals with lower than 13–15% kernel moisture contents, which is required for safe storage (Magan and Aldred, 2007) (Figure 2). Nevertheless, artificial drying is unsurmountablein most cases.

Obeying quality regulations, the recommended drying temperature is less than 65°C for most feed cereals and below 90°C for corn (Hellevang, 2013). Of course, these high drying temperatures will also have on impact on the *Aspergillus* spp., which contaminate grains. *A. flavus* has an outstandingly high heat tolerance in comparison to other fungi with an upper tolerance limit of 40°C (Neme and Mohammed, 2017). Prencipe et al. (2018) also found that while the growth of *A. flavus* was suboptimal above 40°C this relatively high temperature resulted in the most intensive aflatoxin synthesis on chestnut. Hawkins et al. (2005) found that 60°C drying temperature still had no adverse effect on *A. flavus* thriving on corn kernels but raising the temperature up to 70°C significantly decreased fungal infection. Favorable effects of high drying temperature in the restriction of fungal growth were also reported for rice (Hell and Mutegi, 2011).

Unfortunately, the aflatoxin molecules are highly heat-stable as their decomposing temperature is 268–269°C (Peng et al., 2018). As a result, simple drying technologies cannot decrease aflatoxin concentrations significantly in stored grains. On the other hand, elongated high-temperature treatments may have beneficial effects (Lee et al., 2015).

The temperature, kernel moisture content and relative humidity during storage all influence the physiological processes of fungi. As demonstrated, 18 – 19°C temperature and 12 – 13% moisture content were the limiting factors for the growth and activity of the Aspergilli (Villers, 2014; Mwakinyali et al., 2019), although lower temperature (8 – 10°C) may also be permissive for growth and mycotoxin production when the grain moisture content is higher (Mannaa and Kim, 2017). Although these values are accepted widely in good storage practices under continental climatic conditions the relative humidity of grain silos are higher during the cold months, which results in higher water binding by the grains. Nevertheless, the lower temperature hinders increases in microbial activity, and the tolerable water activity is 0.70 for the different *Aspergillus* species (Mannaa and Kim, 2017). It is important to note that ‘hot spots’ can develop in grain heaps because of insects or increased grain physiological activity and the released heat and moisture can support fungal growth. Therefore, maintaining good hygienic practice and controlling the temperature of the grain heaps are adequate and necessary measures during storage (Magan et al., 2003; Peng et al., 2018).

There are several procedures applicable to decrease fungal infection and mycotoxin production in kernels during storage (Table 2). Size separation by sieving and density separation by gravity table are useful measures as the lighter, smaller and broken kernels and the small components of heap may be infected or damaged by fungi and, therefore, they can be starting points for further deterioration. Not surprisingly, their removal significantly decreases aflatoxin contamination (De Mello and Scussel, 2007; Shi et al., 2014; Peng et al., 2018).

**TABLE 2:** Summary of possibilities and examples for the reduction of the aflatoxin content of stored grains.

	**Method**	**Effect**	**References**
Removal	Cleaning and sorting by size and density	Only small Brazil nuts (smaller than 36.6 mm length and 6.3 g weight) contained AFB_1_	De Mello and Scussel, 2007
		Corn particles passed 5.16 mm sieve contained 46 times higher more toxin than the over fraction and lower density kernels contain 50 times higher aflatoxin	Shi et al., 2014
	Sorting by color	UV light, fluorescent and multi spectral analysis can be used to detect contaminated kernels	Pasikatan and Dowell, 2001; Vasishth and Bavarva, 2016; Stasiewicz et al., 2017; Tao et al., 2018
	Removal of contaminated part by dehulling and polishing	Dehulling removed 92% of the initial aflatoxin content from corn kernel	Siwela et al., 2005
		Aflatoxin residuals in corn after crushing and dehulling was almost negligible	Fandohan et al., 2005
		Dehulling decreased AF content of corn by 5.5–70%	Mutungi et al., 2008
		Dehulling and whitening of rice kernel resulted 96% decrease in AF content in polished broken grains and 79% in polished whole kernels	Castells et al., 2007
		Dehulling of corn kernels resulted in 88 and 92% reduction in AFB_1_ and AFB_2_ levels	Matumba et al., 2015
Reduction, destruction	Thermal treatment for a long time	Heating at 100 and 150°C for 90 min decreased the AFB_1_ content of soybean 41.9 and 81.2%, respectively	Lee et al., 2015
	Oxidation by ozone	2.8 and 5.3 mg/l ozone concentration applied for 4 hours resulted 76–84% decrease in AFB_1_ content of poultry feed	Torlak et al., 2016
		66–95% AFB_1_ reduction in peanut, corn and wheat kernel	Ismail et al., 2018
	Irradiation by ionizing and non-ionizing radiation	25 kGy gamma irradiation resulted 43% decrease, microwave heating for 10 min at 1.45 kW resulted 32% decrease, direct solar irradiation for 3–30 h resulted 25–40% decrease in AFB_1_ content of poultry feed	Herzallah et al., 2008
		4, 6, and 8 kGy gamma irradiation doses resulted 15–56% reduction in aflatoxin content for corn, wheat and rice kernels	Mohamed et al., 2015
		5 and 10 kGy irradiation doses resulted in 69.8 and 94.5% decreases in AFB_1_ content, respectively	Markov et al., 2015
		Pulsed light treatment (0.52 J/cm^2^/pulse in spectrum of 100–1100 nm with a xenon flash lamp) resulted 75–90% decreases in AFB_1_ and AFB_2_ contents of rice and rice bran	Wang et al., 2016
		6 and 10 kGy gamma irradiation doses resulted 90 and 95% reduction in AFB_1_, respectively	Serra et al., 2018
		In peanuts, 5–9 kGy gamma irradiation doses result 20–43% decrease in aflatoxins, microwave radiation at 360, 480, and 600 W resulted 59–67% decrease, combined treatments have higher than 95% efficiency	Patil et al., 2019
	Destruction by cold plasma	Hazelnuts, peanuts, and pistachio nuts treated with air gases plasma for 20 min resulted 50% decrease in total aflatoxins, SF_6_ plasma application resulted only 20%reduction	Basaran et al., 2008
		Atmospheric plasma generated with 400–1150 W power for 1–12 min resulted 46–71% decrease in AFB_1_ in peanuts	Siciliano et al., 2016
		High voltage atmospheric cold plasma applied for 1 and 10 min resulted 62 and 82% reduction in AFs levels of corn.	Shi et al., 2017
		Atmospheric and low pressure cold plasma reduced the AFB_1_ content of hazelnut by 72–73%	Sen et al., 2019

Hand sorting based on visible fungal infections is a very useful tool to decrease the aflatoxin B_1_ (AFB_1_) content of corn kernels but obviously this is not a viable option in industrial scale (Matumba et al., 2015). Another possibility is optical sorting because *A. flavus* contaminated corn kernels emit bright greenish-yellowish light when illuminated by UV light enabling separation using suitable optical equipment. Unfortunately, such light emission does not occur in each case and hidden, internal fungi contaminations have no visible effects either. Nevertheless, a sorting method based on the evaluation of red and green light reflectance was also developed to separate aflatoxin containing peanuts and another one for cleaning pecans, based on fluorescence (Pasikatan and Dowell, 2001). It is noteworthy that a low cost multi-spectral analyzer was manufactured to screen single corn kernels at nine distinct wavelengths in the 470 – 1550 nm region for qualitative use (Stasiewicz et al., 2017). Although fluorescent optical techniques have higher sensitivities and specificities than near infrared spectroscopy-based and hyperspectral imaging methods near infrared spectroscopic evaluations seem to have greater capabilities to reveal both aflatoxin and fungal contaminations. Most importantly, these techniques have already been applied in automatic sorters (Tao et al., 2018). Color analyses can be combined easily with other visible properties. For example, the Raspberry Pi optical analytical equipment (Vasishth and Bavarva, 2016) is able to sort peanuts based on their color, size, edge length and area of kernel with more than 40 kg/h sorting capacity. It is foreseeable that recent improvements in computing techniques will open new ways for visual analyses in combating both fungi and their mycotoxins.

Dehulling, the removal of external layers of kernel surface, can be an effective tool to decontaminate grains from toxigenic fungi and significantly decreases the aflatoxin content of grains (Siwela et al., 2005; Peng et al., 2018). This beneficial effect could be improved further by floating and washing before application (Fandohan et al., 2005; Mutungi et al., 2008; Matumba et al., 2015; Hadavi et al., 2017). Polishing rice kernels is also effective to reduce aflatoxin and, hence, more than nine-fold decrease in contamination was recorded (Castells et al., 2007).

Application of ozone during cereal storage is a relatively new method to improve storage conditions, based on the combined antifungal and insecticide effects of this reactive gas (Isikber and Athanassiou, 2015). Importantly, ozone treatments reduce mycotoxin contaminants without any negative effect on the quality of the grains (Tiwari et al., 2010; Zhu, 2018), and eliminate aflatoxins with high efficiency (66–95% of the original toxin concentration) in cereal grains and flours, as well as in soybean and peanut (Torlak et al., 2016; Ismail et al., 2018).

Another physical method to reduce aflatoxin contaminations is irradiation. Several radiation sources have been evaluated thus far and many of them were found to be effective. For example, the advantageous effects of UV in liquid phase (Patras et al., 2017), gamma irradiation in corn (Markov et al., 2015; Serra et al., 2018), in other cereal kernels (Mohamed et al., 2015), in peanuts (Patil et al., 2019) and in poultry feed (Herzallah et al., 2008) have been reported in a number of publications. Direct sunlight was also effective in aflatoxin reduction in poultry feed (Herzallah et al., 2008) and, in addition to exposures to direct light, the applicability of pulsed light has also been tested and evaluated, and it has already been employed in new decontamination technologies (Moreau et al., 2013). Meanwhile exposure to pulsed light was effective in liquid medium (Moreau et al., 2013) pulsed polychromatic light applied with a simple xenon flash lamp also resulted in significant decreases in the aflatoxin content in cereal kernels (Wang et al., 2016).

Cold plasma treatment is another possible physical treatment against pathogens and fungal toxins. Cold plasma is generally a result of atmospheric dielectric discharge, and the effects of pressure (atmospheric or vacuum), air composition, humidity and flow rate, discharging power and treatment time are under continuous evaluation nowadays in different cereals and nuts (Basaran et al., 2008; Siciliano et al., 2016; Shi et al., 2017; Misra et al., 2019; Sen et al., 2019). Cold plasma treatments are cost effective, ecologically neutral and have only a negligible effect on the quality of the grains when compared to classical detoxification methods (Hojnik et al., 2017).

## Fungal Activity and Aflatoxin Production in Silage

Climate change has a major impact on agriculture in many ways and, thereby, many studies have already been published on the effects of climate change on the growth, spread and toxin production of mycotoxigenic fungi on economically important crops (Magan et al., 2011; Paterson and Lima, 2011; Wu et al., 2011; Battilani et al., 2012, 2016).

Aflatoxin contaminations of maize, wheat, etc. have become a major safety issue in the European agricultural industry (Battilani et al., 2016), and aflatoxin producer *Aspergillus* spp. have also been detected in temperate Europe (Dobolyi et al., 2013). As a consequence, mycotoxins including the *Aspergillus*-derived harmful aflatoxins may also contaminate European agricultural products – a foreseeable threat, which we should by no means neglect (Magan et al., 2011; Battilani et al., 2012, 2016; Dobolyi et al., 2013).

Maize silage, one of the most important components in the feeding of dairy cows in Europe and worldwide, can be contaminated by several mycotoxin-producer fungi entering the feed production chain at various stages (Ogunade et al., 2018). Not surprisingly, aflatoxin contaminations can be detected occasionally both before and after ensiling (Storm et al., 2014; Gallo et al., 2015; Ogunade et al., 2018; Peng et al., 2018). Therefore, the rigorous control of the growth of aflatoxigenic fungi is of pivotal importance, if the production of aflatoxin-free silage is to be guaranteed (Borreani and Tabacco, 2010; Ogunade et al., 2018).

Although microaerophilic conditions and low pH, which are typical features of silage fermentations, may prevent the growth of the majority of molds, some species of the genera *Aspergillus*, *Byssochlamys*, *Monascus*, *Penicillium*, and *Trichoderma* are able to survive even under ensiling conditions (Mansfield and Kuldau, 2007; Pereyra et al., 2008). To make things even worse, the aflatoxigenic capacity of the *Aspergillus* section *Flavi* strains derived from silage samples is remarkable. For example, del Palacio et al. (2016) demonstrated that 27.5% of these strains produced AFB_1_, 17.5% of them aflatoxin G_2_ (AFG_2_) and 10% synthesized aflatoxin G_1_ (AFG_1_). Interestingly, only 5% of the strains produced AFB_2_ (del Palacio et al., 2016). In another study concomitantly performed in Pakistan (Sultana et al., 2017), *A. niger*, *A. flavus, A. fumigatus, A. ochraceous*, and *A. terrus* were identified in both fresh fodder and corn silage. Importantly, the authors also found AFB_1_ in 37.5% of the fresh fodder and in 41.7% of the corn silage samples with average AFB_1_ concentrations of 9.5 and 8.4 μg/kg, respectively, meanwhile AFB_2_ was present in only two samples (1.2 and 1.3 μg/kg), and none of the analyzed samples was contaminated by AFG_1_ or AFG_2_ (Sultana et al., 2017). In Southern Brazil, aflatoxigenic *A. parasiticus* and *A. nomius* strains have been detected in the tested silage and concentrated feed samples (Variane et al., 2018).

Considering the world-wide occurrence of aflatoxin contaminations (Table 3), AFB_1_ has been reported in corn silage in Argentina (González Pereyra et al., 2008, 2011), in Brazil (Keller et al., 2013; Schmidt et al., 2015), and in France (Richard et al., 2009). Total aflatoxin contaminations have also been determined in silage samples collected in Iran (Hashemi et al., 2012) and in Uruguay (del Palacio et al., 2016).

**TABLE 3:** Worldwide occurrence of aflatoxins in silage.

**Country**	**Mycotoxin**	**Sample**	**No. of samples**	**No. of positive sample (Incidence%)**	**Mean concentration (μg/kg)**	**Range (μg/kg)**	**References**
Argentina	AFB_1_	Corn silage	35	6(17.0%)	–	1.4 – 155.8	González Pereyra et al., 2008
Argentina	AFB_1_	Trench silo	43	6(14.0%)	–	1.0 – 190.0	González Pereyra et al., 2011
Argentina	AFB_1_	Silo bag	35	19(54.3%)	–	5.8 – 47.4	González Pereyra et al., 2011
Brazil	AFB_1_	Corn silage	116	15(13.0%)	33.0	2.0 – 61.0	Keller et al., 2013
Brazil	AFB_1_	Corn silage	327	3(0.9%)	3.0	1.0 – 6.0	Schmidt et al., 2015
France	AFB_1_	Corn silage	–	–	28.0	7.0 – 51.3	Richard et al., 2009
Iran	Total AF	Silage	42	7(16.7%)	1.24	1.1 – 27.3	Hashemi et al., 2012
Uruguay	Total AF	Wheat silage	220	–	17.0	6.1 – 23.3	del Palacio et al., 2016

*–, not evaluated data.*

## Microbial Biocontrol and Microbial Detoxification Products for Mycotoxin Mitigation in Animal Husbandry

In recent decades, several feasible and cost-effective strategies have entered the market aiming to mitigate the effects of feed mycotoxin contamination in animal husbandry, especially in the dairy industry. Technologies to reduce the incidence of mold and mycotoxin contaminations of silages can be employed in one of the three main phases (preharvest, harvest, ensiling) of silage production. During the preharvest phase, the appropriate agronomic practices may rely on (i) the use of crop varieties or hybrids, which are resistant to fungal infections, (ii) the application of pesticides and fungicides, (iii) adequate management of weeds and crop residues, (iv) the use of appropriate crop rotation, tillage, fertilization and irrigation and (v) the application of biocontrol agents, e.g., bacteria, yeasts, or atoxigenic strains of *A. flavus* or *A. parasiticus* (Gallo et al., 2015; Pfliegler et al., 2015; Ogunade et al., 2018; Peng et al., 2018). During the harvest phase, the most important factors that should be taken into consideration are proper harvest timing (maturity stage) and cutting height (to minimize soil contamination), as well as immediate storage of harvested feeds (Gallo et al., 2015; Ogunade et al., 2018; Peng et al., 2018).

Pre-harvest biocontrol microbes represent a promising and already widely applied method to lower mycotoxin risks in food and feed by protecting plants from pathogens and inhibiting the growth of molds during postharvest conditions. They both reduce economic loss caused by fungal infections and lower toxin levels in products (e.g., Pfliegler et al., 2015). Biocontrol agents compete for nutrients and space, may secrete antifungals or even parasitize molds, and can also stimulate host plant resistance (Liu et al., 2013) and, thereby, they mitigate the risk of plant infections and their undesirable consequences. Regarding Aspergilli infection and aflatoxin contamination, non-aflatoxigenic biocontrol *Aspergillus flavus* strains are most commonly applied to crops (Ehrlich, 2014; Weaver and Abbas, 2019), while biocontrol yeasts species are also effective, such as the 2-phenylethanol producing *Wickerhamomyces anomalus* (Hua et al., 2014). These biocontrol agents are mostly applied to protect plants directly used in food production but may exert their effects on plant parts that are to be ensiled for feed production concomitantly.

In the ensiling phase, attention must be payed to adequate particle size, proper silo size, immediate rapid filling, proper compaction, complete sealing (to maintain strictly anaerobic conditions), and the use of acid-based additives or microbial inoculants, e.g., lactic acid bacteria (Gallo et al., 2015; Ogunade et al., 2018; Peng et al., 2018). Some specific strains in the *Lactobacillus* (*L. buchneri, L. fermentum, L. hilgardii, L. plantarum, L. reuteri, L. rhamnosus*), *Lactococcus* (*L. lactis*), *Leuconostoc*, and *Pediococcus* (*P. pentosaceus*) genera can inhibit or can even prevent completely the growths of various mycotoxigenic molds and their mycotoxin productions as well (Dalié et al., 2010; Cavallarin et al., 2011; Queiroz et al., 2012; Dogi et al., 2013; Ahlberg et al., 2015; Ma et al., 2017; Gallo et al., 2018; Zielińska and Fabiszewska, 2018; Ferrero et al., 2019). It is noteworthy that there is a wide spectrum of environmental factors which influence the antifungal activity of LAB, including the type of the matrix and culture medium, the availability of nutritional compounds, the incubation time and temperature (Dalié et al., 2010; Ahlberg et al., 2015; Leyva Salas et al., 2017). In addition, some biological (e.g., the natural microbiota), and chemical (e.g., pH, water activity) parameters will also affect the antifungal activity in a species-specific manner (Dalié et al., 2010; Ahlberg et al., 2015; Leyva Salas et al., 2017). Species- and strain-specific factors are noteworthy, for example both *L. rhamnosus* and *L. plantarum* were efficient against *A. parasiticus* only *L. rhamnosus* reduced the AFB1 levels produced by *A. parasiticus* (Dogi et al., 2013). Quite unexpectedly, the *A. parasiticus* – *L. plantarum* interaction even stimulated aflatoxin B_1_ production, which makes the use of *L. plantarum* undesirable as a silage inoculant.

In another study, a mixture of *P. pentosaceus* and *L. buchneri* reduced the adverse effects of rust infestation during ensiling and also decreased aerobic spoilage and aflatoxin production in maize silages with high levels of southern rust infestation (Queiroz et al., 2012). Importantly, *L. buchneri* increased the aerobic stability of the silage as well (Cavallarin et al., 2011). Inoculation of corn silage with a combined inoculant of *L. buchneri* and *Lactococcus lactis* improved the aerobic stability of the silage, and the higher silage density increased the stability further (Gallo et al., 2018). The interaction of *L. buchneri*, *L. reuteri*, *L. plantarum*, and *L. fermentum* strains reduced the AFB_1_ level, improved the stability and, furthermore, the microbiological and chemical purity of maize silage (Zielińska and Fabiszewska, 2018). In a most recent study by Ferrero et al. (2019), the authors examined the effect of *L. buchneri*, *Lactobacillus hilgardii*, and their combination on *A. flavus* contaminants and their aflatoxin production in maize silage. The results showed that the inoculation of corn silage with *L. buchneri* and *L. hilgardii* increased the aerobic stability and delayed the beginning of aerobic microbial degradation of maize silage, and indirectly reduced the risk of *A. flavus* emergence and aflatoxin B_1_ level after silage opening.

Ma et al. (2017) examined the AFB_1_ binding capacity of various silage bacteria including *L. plantarum*, *L. buchneri*, *P. acidilactici*, and *P. pentosaceus* and found that high concentration of silage bacteria could bind the AFB_1_ content of maize silage but population, strain, viability, and medium acidity have all affected the efficacy of binding.

Antifungal compounds produced by LAB also reduce the mycotoxin production of molds (Ahlberg et al., 2015). These LAB-produced compounds cover organic acids (e.g., acetic, lactic, and propionic acid), carboxylic acids, phenolic compounds, including phenolic acids (gallic acid, tannins, benzoic acids, phenyllactic acid, hydroxyphenyllactic acid), fatty acids (caproic acid, decanoic acid, 3-hydroxydecanoic acid, coriolic acid, ricinoleic acid), volatile compounds (e.g., diacetyl, acetoin), cyclopeptides [*e.g.*, cyclo(Phe-Pro), cyclo(L-Leu-L-Pro), cyclo(L-Met-L-Pro), cyclo(L-Tyr-L-Pro)], hydrogen peroxide, ethanol, reuterin, and proteinaceous compounds (Dalié et al., 2010; Li et al., 2012; Crowley et al., 2013; Le Lay et al., 2016; Leyva Salas et al., 2017).

Considering the mechanisms of actions of these antifungals, the dissociated forms of organic acids can decrease the intracellular pH within the cells, can increase the permeability of the cytoplasmic membrane, and finally can lead to the death of the fungal cells (Leyva Salas et al., 2017). In addition, H_2_O_2_ oxidizes directly the cellular proteins and the lipid components of the cellular membranes (Dalié et al., 2010). Nevertheless, the mechanisms of the antifungal actions of hydroxy fatty acids and proteinaceous compounds have remained yet to be elucidated (Dalié et al., 2010).

Silage decontamination may also be applied if measures to avoid contamination were proven ineffective. Such strategies are primarily based on the adsorbents. Advantages of using adsorbent feed additives over decontamination of the final product, e.g., milk, are their safety and inexpensiveness, and that they may simply be mixed into animal feed to achieve the desired effect. These products may lower the bioavailability of mycotoxins and can help to decrease toxic effects, as well as the amount of toxin detectable in the final product (meat or milk). Such strategies may involve the use of live microbial (LAB or yeast) cultures (usually termed microbial enterosorption, biosorption), microbial or plant extracts, other organic/inorganic materials such as activated carbons or charcoals, hydrated sodium calcium aluminosilicates, and various clay-based products (Kutz et al., 2009; Giovati et al., 2015). LAB can not only inhibit the growth of molds but are also able to bind aflatoxins in different matrices (Table 4; Ahlberg et al., 2015; Muck et al., 2018), thereby reducing the health risks of aflatoxins. Environmental conditions have a great impact on the aflatoxin binding capabilities of LAB (Dalié et al., 2010; Ahlberg et al., 2015; Ma et al., 2017), which is highly species-specific (Gomah et al., 2010; Dogi et al., 2013; Ahlberg et al., 2015). Some studies demonstrated that non-viable LAB cells had better binding capability for aflatoxin than viable LAB cells (Ahlberg et al., 2015; Damayanti et al., 2017; Ma et al., 2017). On the contrary, Liew et al. (2018) reported on a higher binding efficiency by living cells. Regardless of alive or dead bacterial cells, the aflatoxin binding seems to be reversible and the bound mycotoxins are released slowly over time (Verheecke et al., 2016).

**TABLE 4:** Antifungal activity of lactic acid bacteria (LAB).

**LAB**	**Strain**	**Effect**	**References**
*Lactobacillus buchneri*	NCIMB 40 788	Decreased mold count, decreased AFB_2_ and increased aerobic stability of the silage	Cavallarin et al., 2011
*Lb. buchneri*	40788	Decreased the population of spoilage fungi, and aflatoxin production in silages	Queiroz et al., 2012
*Lb. buchneri*	R1102	Bound AFB_1_	Ma et al., 2017
*Lb. buchneri*	LB1819	Enhanced the fermentation and aerobic stability of maize silage	Gallo et al., 2018
*Lb. buchneri*	A KKP 2047 p	Reduced mold count and decreased AFB_1_ amount	Zielińska and Fabiszewska, 2018
*Lb. buchneri*	NCIMB 40788	Reduced the risk of *Aspergillus flavus* outgrowth and AFB_1_ production after silage opening	Ferrero et al., 2019
*Lactobacillus fermentum*	N KKP 2020 p	Reduced mold count and decreased AFB_1_ amount	Zielińska and Fabiszewska, 2018
*Lactobacillus hilgardii*	CNCM I-4785	Reduced the risk of *Aspergillus flavus* outgrowth and AFB_1_ production after silage opening	Ferrero et al., 2019
*Lactobacillus plantarum*	RC009	Reduce *Aspergillus parasiticus* growth rate	Dogi et al., 2013
*Lb. plantarum*	PT5B	Bound AFB_1_	Ma et al., 2017
*Lb. plantarum*	K KKP 593 p, S KKP 2021 p	Reduced mold count and decreased AFB_1_ amount	Zielińska and Fabiszewska, 2018
*Lactobacillus reuteri*	M KKP 2048 p	Reduced mold count and decreased AFB_1_ amount	Zielińska and Fabiszewska, 2018
*Lactobacillus rhamnosus*	RC007	Reduce *Aspergillus parasiticus* growth rate	Dogi et al., 2013
*Lactococcus lactis*	O224	Enhanced the fermentation and aerobic stability of maize silage	Gallo et al., 2018
*Pediococcus pentosaceus*	12455	Decreased the population of spoilage fungi and aflatoxin production in silages	Queiroz et al., 2012
*Pediococcus acidilactici*	R2142, EQ01	Bound AFB_1_	Ma et al., 2017

Based on various microbe species, sources, manufacturers, and formulations, live yeast products include several categories: yeast probiotics, *Saccharomyces cerevisiae* fermentation products (SCFP), dried yeast products (DY or DYP), brewery yeasts (BY), and active dry *S. cerevisiae* (ADSC) (Pizzolitto et al., 2012; Poppy et al., 2012; Gonçalves et al., 2017). Compared to live bacteria-based products, these yeast products are considered and employed as direct feed additives in most cases and are not applied at the ensiling phase (Giovati et al., 2015). Some bacterial species, e.g., *Nocardia corynebacteroides* (NC) are also added as direct feed additives for poultry (Tejada-Castañeda et al., 2008). Microbe-derived feed additive products are also based on yeasts, and include autolyzed yeast (AZ), inactivated yeast cells (IY), distillery yeast sludge, and yeast cell wall (YCW) products (Gonçalves et al., 2017; Plaizier et al., 2018).

Live yeast or bacterial cells intended to colonize the gastrointestinal tract (GIT) of humans, or in some cases, poultry or laboratory rodents, are occasionally termed probiotics (Śliżewska and Smulikowska, 2011; Pizzolitto et al., 2012; González Pereyra et al., 2014). However, especially in the case of ruminants, the use of live cells may not necessarily result in gastrointestinal colonization. The rumen’s own microbiota is also to be taken into account, as it can contribute to aflatoxin detoxification and degradation (e.g., biotransformation to aflatoxicol) (Upadhaya et al., 2009; Jiang et al., 2012). Aflatoxin B1 is absorbed in the rumen mainly at acidic pH (Pantaya et al., 2014), and the degradation of aflatoxins in rumen depends on both the animal species and feed type (Upadhaya et al., 2009). However, it must be noted that rumen colonization by *A. flavus* has also been recorded, leading to toxin production in rumen liquor (Nidhina et al., 2017).

**FIGURE 3:**
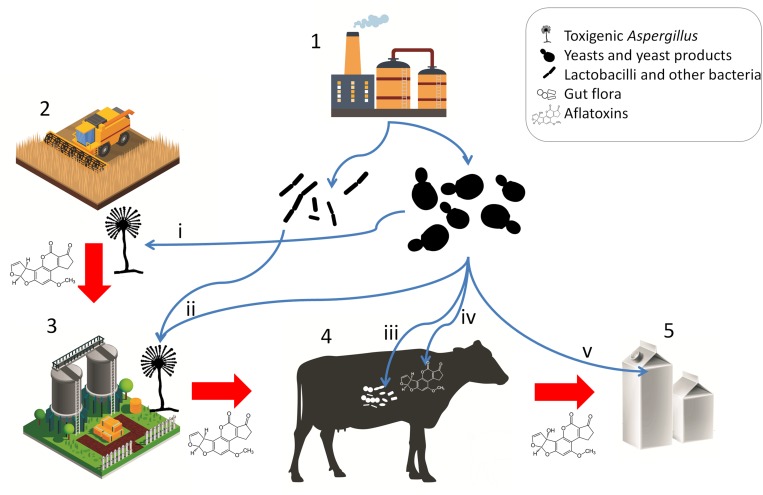
Microbial products for mycotoxin mitigation in animal husbandry and their applications. Red arrows represent potential carry-over of mycotoxins or toxigenic *Aspergilli*. Blue arrows represent applications of microbes and microbe-derived products. (1) Fermentation and animal feed supplement industries; (2) crop production; (3) preparation and storage of silage and other feedstuffs; (4) livestock; (5) product; (i) pre-harvest biocontrol; (ii) antagonism in silage and feed; (iii) host gut microbiota and immune modulation, probiotic effect; (iv) enterosorption; (v) bioadsorption from product (milk). [Stock image credits: Freepik, macrovector, and vectorpocket].

The products SCFP, DY, BY, and ADSC consist of yeast cells, the nutrient medium on which the yeasts were grown, and the metabolites produced by the yeasts and have been shown to increase DMI, milk yield, as well as milk fat and protein yield in lactating dairy cows (Poppy et al., 2012). However, these positive effects are attributed to adsorption of toxic substances and the modulation of the gut (prokaryote) microbiota, not to long-term gut colonization by the yeasts. Yeasts in fact are thought to play a negligible role in the microbiome of ruminants, although they may survive gastrointestinal conditions and retain their aflatoxin B_1_ binding ability under gastrointestinal conditions (Dogi et al., 2011). Various studies have shown the effects of these live yeast products on the microbiota of the cows, however, uncovering the underlying mechanisms and a holistic understanding of dairy cow gastrointestinal health still requires further research (Zhu et al., 2017; Huebner et al., 2019). Interestingly, YCW has also been shown to positively modulate the gut health in broiler chicken challenged with AFB_1_ or with *Clostridium* infection (Liu et al., 2018). These observations raise the possibility that yeast products, whether live or not, generally contribute to animal health both as bioadsorbents and as modulators of the gut prokaryote microbiota, as well as the immune status of the animal. Such positive effects may not only prevent toxicosis but result in increased feed intake and production (Pasha, 2008). In poultry feedstuff, *S. cerevisiae* strains have been tested and made commercially available as a probiotic microbe. It must be noted though, that the intended effect of the yeasts is not necessarily gut colonization and microbiome modulation, but aflatoxin adsorption (Śliżewska and Smulikowska, 2011; Pizzolitto et al., 2012), a role, which yeasts can effectively fulfill. The applications of microbes and microbial products for mycotoxin risk mitigation are summarized in Figure 3.

Yeast cell wall β-D-glucans, glucomannans and mannan-oligosaccharides are responsible for the mycotoxin binding abilities of these products (Pfliegler et al., 2015). Some purified cell wall components have been tested in animal husbandry, such as mannan-oligosaccharides supplemented into the diet of Japanese quails affected by aflatoxicoses (Oguz and Parlat, 2005). However, no direct correlation between the amount of individual components and toxin binding are evident (Joannis-Cassan et al., 2011). Structural integrity and amount of the yeast cell wall is crucial in binding efficacy, while viability is not: heat-treatment can even increase adsorption capacity (Bueno et al., 2007; Joannis-Cassan et al., 2011). Toxin binding can reach saturation rapidly and is reversible, and mycotoxins are not modified chemically during the process (Bueno et al., 2007). It must be noted that some yeasts (reviewed by Pfliegler et al., 2015) and bacteria (Wang Y. et al., 2018) are known to be able to enzymatically degrade mycotoxins if applied in viable form.

A novel approach for the microbiological detoxification of animal feed is the screening of isolates from various environmental sources (Intanoo et al., 2018), instead of using the most widespread species, *S. cerevisiae*. Various bacteria and yeasts may exhibit toxin-binding or even toxin-degrading abilities, as well as biocontrol effects on toxigenic molds (Pfliegler et al., 2015) and these may be directly applied to supplement animal feed (Intanoo et al., 2018). Novel yeast species in this field include members of the genera *Kluyveromyces* and *Pichia*, both related to the widely used *Saccharomyces*. *P. kudriavzevii* has been successfully applied as a bioadsorbent feed additive to ameliorate the negative effects of AFB_1_ contamination on broiler chicken performance (Magnoli et al., 2017). Novel isolates of *K. marxianus* have also been proposed as bioadsorbents based on *in vitro* characterization (Intanoo et al., 2018). However, Battacone et al. (2009) found no evidence for AFB_1_ detoxification in ewes fed with *Kluyveromyces lactis* DYP, highlighting the need for rigorous testing of novel strains in different setups and with multiple animal species.

Apart from novel microbial strains, combined treatments of microbial and inorganic products constitute a promising strategy in ameliorating mycotoxin contamination. Recently, Jiang et al. (2018) found that both dietary clay and clay + SCFP reduced transfer of dietary AFB_1_ to milk as well as milk aflatoxin M_1_ (AFM_1_) concentration, while the combined treatment was the only one that also prevented the decrease in milk yield caused by AFB_1_. Thus, the potent adsorbing capability of inorganic products may act synergistically with the adsorbent, gut health-promoting and immunomodulatory effects of yeast products.

## Microbial Detoxification Products to Counteract Aflatoxin Contamination in Dairy Products

Some studies have explored microbial aflatoxin decontamination strategies in dairy products, taking advantage of the high efficacy and relative ease of utilizing LAB and yeast, recently been reviewed by Assaf et al. (2019). Briefly, such microbial decontamination approaches rely on heat-killed or immobilized cells, and promising results were obtained when both LAB and yeasts were applied simultaneously. Heat-treatment of bacterial cells was found to improve binding capabilities in some studies (Pierides et al., 2000; Bovo et al., 2015; Assaf et al., 2018), while no such effect was reported by Kabak and Var (2008). Bacteria tested in the aforementioned studies include members of the genera traditionally considered probiotics and/or important in food production, as *Bifidobacterium*, *Lactobacillus*, or *Pediococcus*, and oddly, a potential pathogen, Enterococcus.

In UHT skim milk, both LAB and yeasts showed promising results (Corassin et al., 2013), and the binding of toxins to microbial cell walls was shown to be rapid, enabling short incubation times in potential industrial applications. Yeasts of the genera *Saccharomyces* and *Kluyveromyces* have been tested by Abdelmotilib et al. (2018), where the higher efficacy of heat-killed cells was also demonstrated for yeasts.

There are certain limitations on applying yeasts and bacteria for the decontamination of dairy products (Assaf et al., 2019), such as the need for their subsequent removal, reversibility of binding, or even legislations on tolerated number of live or dead microbial cells in products. Nevertheless, the high toxin binding capability and the safety of heat-killed cells toward consumers compared to chemical methods makes microbial decontamination a promising strategy.

## Aflatoxin Metabolism in Livestock

**FIGURE 4:**
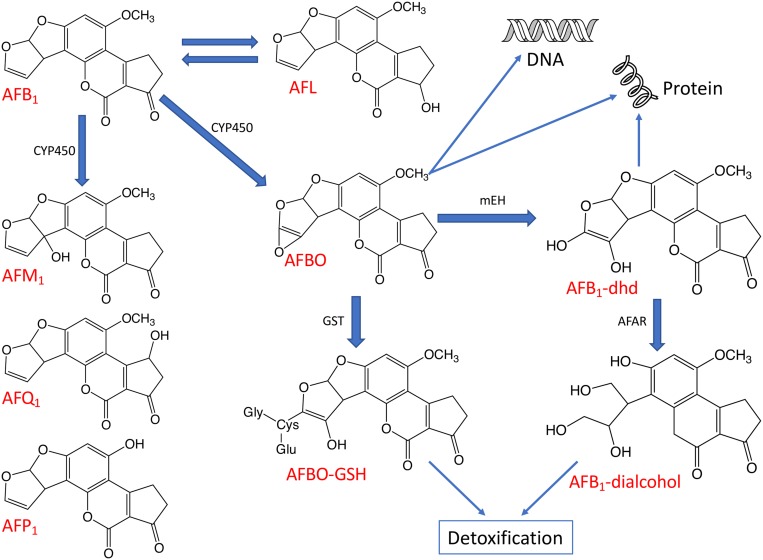
The major metabolic pathways of AFB_1_. The aflatoxin derivatives presented here include aflatoxin B_1_ (AFB_1_), aflatoxin M_1_ (AFM_1_), aflatoxin Q_1_ (AFQ_1_), aflatoxin P_1_ (AFP_1_), AFB_1_-8,9-epoxide (AFBO), AFB_1_-8,9-dihydrodiol (AFB_1_-dhd) and aflatoxicol (AFL). Some enzymes taking part in the biotransformation and detoxification of aflatoxins are also indicated including cytochrome P450 (CYP450), glutathione-*S*-transferase (GST), microsomal epoxide hydrolase (mEH), and aflatoxin-aldehyde reductase (AFAR).

The toxicity of AFB_1_ is strictly related to the bioactivation and detoxification pathways operating animals *in vivo* (Figure 4). Indeed, AFB_1_ is a “pro-carcinogen” that is activated biologically by cytochrome P450 (CYP450), a microsomal enzyme of phase I detoxification (oxidation) to the extremely reactive and electrophilic AFB_1_-8,9-epoxide (AFBO). This harmful AFB_1_ derivative is able to covalently bind to macromolecules such as DNA and proteins, thereby forming adducts, which cause acute and chronic cytotoxicity, DNA mutations and eventually expressing carcinogenic activity (Diaz et al., 2010; Deng et al., 2018).

Moreover, AFBO can be hydrolyzed to AFB_1_-8,9-dihydrodiol (AFB_1_-dhd) by an epoxide hydrolase. AFB_1_-dhd is able to react with proteins causing cytotoxicity or, alternatively, AFBO can be metabolically detoxified *via* conjugation with glutathione (GSH) by glutathione S-transferase (GST), a phase II detoxification enzyme. This pathway is considered as one of the main routes of AFBO detoxification (Diaz et al., 2010; Deng et al., 2018). Microsomal epoxide hydrolase (mEH) and aflatoxin-aldehyde reductase (AFAR) can also transform reactive AFB_1_ to AFB_1_-dialcohol, a real detoxified AFB_1_ derivative, which can be excreted in urine (Guengerich et al., 2001; Deng et al., 2018).

It is important to note that several isoenzymes belonging to the CYP450 supergene family metabolize AFB_1_ through oxidative reactions, producing various metabolites with different carcinogenic potential.

In addition to the highly reactive and toxic AFBO, the main AFB_1_ metabolic pathways described in animals can also give rise to relatively less toxic metabolites such as aflatoxicol (AFL) by ketoreduction or AFM_1_ by hydroxylation and non-toxic metabolites such as AFB_2__a_ or aflatoxin Q_1_ (AFQ_1_) by hydroxylation or aflatoxic P_1_ (AFP_1_) by demethylation (Figure 4; Dohnal et al., 2014; Deng et al., 2018).

Current literature data indicate that the rates of the bioactivation and detoxification of AFB_1_ contribute greatly to the manifestation of its toxic effects. Furthermore, the metabolism of aflatoxins shows considerable interspatial differences and also significant variations among individuals belonging to the same species, depending for example on the age (Dohnal et al., 2014).

In conclusion, the largely different sensitivities of different animal species to the toxic effects of aflatoxins could be explained mainly with the remarkable variability of the metabolic pathways and enzymes that contribute to the bioactivation and detoxification of aflatoxins (Dohnal et al., 2014).

### Poultry

Poultry are generally very sensitive to AFB_1_ and adverse health effects have been reported in turkeys, quail, chickens, and ducks but there is a great variability in species-specific sensitivities to aflatoxins (Klein et al., 2000; Diaz et al., 2010; Rawal et al., 2010). Several toxicological studies pointed at the existence of a sensitivity scale starting from the less resistant young duck and turkey, followed by quails, up to the more resistant chickens. Obviously, species-specific differences in the AFB_1_ biotransformation pathways, e.g., in AFB_1_ modifying hepatic microsomal enzymes, could explain the varying susceptibilities of the species (Lozano and Diaz, 2006). It has been reported in some works that the microsomal liver fractions produced only AFBO in avian species (Figure 4), unless these animals were stimulated with CYP450 inducers (Lozano and Diaz, 2006). However, the ability of poultry species to metabolize AFB_1_ to AFM_1_ was reported in other works, in which the AFM_1_ was detected in different tissues (Madden and Stahr, 1995; Wang H. et al., 2018). Lozano and Diaz (2006) reported that turkey microsomes produced 1.8–3.5 times more AFBO than quail and chicken microsomes. Furthermore, Diaz et al. (2010) suggested that the higher resistance of chicken to AFB_1_ in comparison to quail could be due to a lower activation rate of AFB_1_ to AFBO in chicken and also to a lower affinity for AFB_1_ of the chicken microsomal enzymes.

The high susceptibility of poultry to AFB_1_ appears to be a consequence of both the high activity of phase I microsomal detoxification enzymes to form AFBO, and to a low GST efficiency as well to conjugate AFBO with GSH (phase II detoxification). Some works reported that the partial or complete lack of GST-dependent detoxification of AFBO was the major reason for the exceptionally high susceptibility of poultry including turkeys to AFB_1_ (Klein et al., 2000; Rawal et al., 2010).

Another metabolic pathway that may contribute to the extreme susceptibility of poultry to aflatoxins could be the reduction of AFB_1_ to AFL *via* cytosolic reductase because the cytosolic metabolite AFL is produced in larger quantities in turkey and duck than in quail and chicken. This fact underlines that the formation of AFL cannot be regarded as a real detoxification pathway in these birds, moreover, microsomal dehydrogenase may oxidize AFL back to AFB_1_, increasing the physiological half life of AFB_1_ (Lozano and Diaz, 2006). Other aflatoxin metabolic pathways may also be involved in the manifestation of the high cytotoxicity of AFB_1_ in poultry species.

Furthermore, it has also been reported that AFB_1_ susceptibility correlated with age in both turkeys and broiler chickens. When livers obtained from 9, 45, and 61 day-old turkeys were compared, microsomes from younger birds were more active in AFB_1_ bioactivation than those from older ones (Klein et al., 2002). Moreover, Wang H. et al. (2018) underlined the efficient bioactivation of AFB_1_ by CYP enzymes and the deficient detoxification by GST enzymes in younger 7-day old broilers.

Aflatoxin residues were detected in various tissues mainly in liver, kidney, the organs where AFB_1_ is metabolized, but also in reproductive organs, in gizzard, breast and in legs (Herzallah et al., 2014). The metabolites AFB_1_, AFQ_1_, and AFL were excreted as such or as glucuronyl conjugates from bile in feces (Yunus et al., 2011). Some of these metabolites (AFM_1_ and AFL) have been found in liver, kidneys and thigh muscles (Micco et al., 1988). The concentrations of AFB_1_ residues decreased in the livers and muscles of all the birds after the suspension of mycotoxin feeding, and the elimination of AFB_1_ from tissues was faster in older than in younger birds (Yunus et al., 2011). The dietary exposure to aflatoxin of hens, even at low concentrations, may also cause contamination of eggs. AFB_1_ residues appeared in eggs after 5 days following the administration of AFB_1_ contaminated feedstuffs, and they accumulated in line with protracted feeding with contaminated grain (Hassan et al., 2012). However, the amount of mycotoxin contaminants was below 0.1% of the AFB_1_ intake owing to the AFB_1_ metabolism in the birds. Few works also demonstrated the presence of hydroxylated AFB_1_ derivatives (AFM_1_ and AFQ_1_) in eggs (Anfossi et al., 2015).

### Pigs

Pigs are considered relatively susceptible to AFB_1_. Tulayakul et al. (2006) studied AFB_1_ metabolism in liver of different species in relation to the susceptibility to the toxic effects. The piglet’s liver showed a relatively lower cytosolic GST activity to convert AFB_1_-epoxide to AFB_1_-glutathione conjugate product, thus favoring the formation of AFB_1_-DNA adducts.

The metabolism and tissue distribution of AFB_1_ in pigs were studied by Lüthy et al. (1980), and the major excretory route was found to be the feces (51–65% of the dose administered) but also urine was also an important excretory route. Actually, both AFM_1_ and AFB_1_ were detected in pig urine samples and AFM_1_ was always found at higher concentrations in all studies (Thieu and Pettersson, 2009). In fact, urinary AFB_1_ and AFM_1_ are often used as biomarkers for aflatoxin exposure in pigs.

### Ruminants

Ruminants are generally more resistant to the toxic effects of mycotoxins than monogastric animals, which could be explained mainly by AFB_1_ degradation or bioconversion by rumen microorganisms. Controversely, some studies reported on that aflatoxins were generally poorly bioconverted in the rumen, with an overall decrease of only 10% (Westlake et al., 1989). Moreover, AFB_1_ was incubated with intact rumen fluid or fractions of rumen protozoa and bacteria from sheep and cattle in the presence or absence of milled feed and the result clearly indicated that rumen fluid had no effect on AFB_1_ (Kiessling et al., 1984). Another study showed that AFB_1_ metabolism in rumen fluid was influenced by the animal species and the type of feed. In fact, rumen microbes from Korean native goats exhibited a greater degradation capacity for AFB_1_ in comparison to Holstein steers. These diverging observations might be the consequence of varying rumen microbe profiles (Upadhaya et al., 2009).

AFM_1_ is the most prominent metabolite formed in bovine hepatocytes within the first hours of incubation whereas AFB_1_-dhd becomes determinative after a prolonged incubation. These two metabolites are mainly formed by CYP1A and CYP3A hepatic monooxygenase activities (Kuilman et al., 2000). According to Larsson et al. (1994), several extrahepatic tissues of sheep can also bioactivate AFB_1_ very efficiently and can conjugate the bioactivated AFB_1_ with GSH as well.

Following the channeling of AFB_1_ in ruminants, the ingested aflatoxins may be degraded, at least in part, to AFL, AFM_1_ and many other hydroxylated metabolites by certain rumen microbes or may be sequestered by some rumen fluid components such as chlorophyllin structures as well as bacterial and yeast cell walls (Gallo et al., 2015). The remaining fraction is rapidly adsorbed in the gastro-intestinal tract by passive diffusion and then is extensively metabolized in the liver to AFM_1_, which enters the systemic circulation or is conjugated to glucuronic acid, and afterward excreted *via* bile, urine or milk (Kuilman et al., 2000; Rodrigues et al., 2019). Obviously, different levels of feed contamination may lead to different carry-over rates, which are also influenced by other physiological factors such the health status of animals including the status of the liver and its enzymatic activities. AFB_1_, AFM_1_ and AFL have been detected in liver, kidney and muscle tissue of bovine (Kuilman et al., 1998). AFM_1_ is excreted *via* urine at a greater extent than through milk but the physiological factors regulating the relative uptake by kidneys and mammary glands are still unknown (Rodrigues et al., 2019). AFM_1_ has been detected in both the milk and urine of cattle and dairy ewes 6 h after AFB_1_ ingestion (Helferich et al., 1986; Battacone et al., 2003), and its concentration decreased rapidly after withdrawal of aflatoxin from diets (Rodrigues et al., 2019). Fecal excretion of FB_1_ results from a lack of absorption by the GIT or a highly efficient elimination by the biliary system in the form of conjugated metabolites (Yiannikouris and Jouany, 2002; Jouany et al., 2009).

Goats were administrated with [^14^C]-AFB_1_, and urine, milk and feces were collected after 120 h. AFM_1_ was found in milk at the highest concentration meanwhile AFQ_1_ and AFL were found only in trace quantities in milk (Helferich et al., 1986). Other studies on goats also indicated that the absorption of AFB_1_ in the GIT of adult ruminants was very fast, as was its hydroxylation to AFM_1_ and release into the blood (Battacone et al., 2012). The short interval between AFB_1_ administration and the detection of its metabolite in milk confirmed that the absorption of the toxin took place already in the rumen in goats.

## Aflatoxins in Foods of Animal Origin

Aflatoxins are generally considered as the most important mycotoxins due to their carcinogenic properties, their persistence in food commodities once formed, and the wide range of food commodities that may be contaminated by them (Fink-Gremmels and van der Merwe, 2019). Aflatoxins contaminating feeds pose a direct threat to livestock health and, indirectly, also affect human nutrition and health by reducing livestock productivity and *via* transfer from feed to foods of animal origin, namely milk, meat and eggs, even if milk is the only food of animal origin with relevant aflatoxin carry-over (Frazzoli et al., 2017).

Aflatoxins, particularly AFM_1_, are of public health concern because they are efficiently excreted into milk, even if they may also contaminate other foods of animal origin at low levels and, therefore, the associated risks are considered to be minor (Fink-Gremmels and van der Merwe, 2019). Not surprisingly, many countries have set maximum levels of aflatoxins (AFB_1_ or total aflatoxins, AFM_1_) in food commodities and animal feeds, with the main aims to protect animal health and to prevent aflatoxin contamination of animal-derived foods. This review does not provide a systematic overview on aflatoxins in foods of animal origin but summarizes the discussions on the potential public health concerns specifically related to aflatoxins residues in these food commodities. In livestock animals, the best estimate transfer factors for mycotoxins in kidney, liver, muscle, fat, milk and egg were reported by MacLachlan (2011), and they clearly showed that no significant residues coming from aflatoxin contaminants of livestock feed are present in meat and eggs.

In the case of human dietary exposure from dairy products, aflatoxins are considered the most important mycotoxins and, based on data belonging to Food and Feed Safety Alert, 93% of the overall mycotoxin notifications referred to aflatoxins, whereas dioxins, dioxin-like polychlorinated biphenyls and AFM_1_ were the most frequently reported chemical issues in dairy products (van Asselt et al., 2017). When ruminants were fed with contaminated feed, the AFB_1_ consumed by the animals was partly degraded by the forestomach before reaching the circulatory system, and the remaining part was transformed by the liver into monohydroxy derivative forms, mainly to AFM_1_, and, in smaller quantities, also to AFM_2_, AFM_4_ and AFL. Afterward, AFM_1_ was secreted into the milk through the mammary glands (Frazzoli et al., 2017). AFM_1_ has only from 2 to 10% of the carcinogenic potency of AFB_1_ but it possesses the same liver toxicity. The ability of ruminants to convert the AFB_1_ ingested with feedstuff to AFM_1_ and to excrete this derivative in milk varies within broad limits in large and small ruminants and ranges between 0.35 and 3% in cows, 0.018 and 3.1% in goats and between 0.08 and 0.33% in sheep (Virdis et al., 2014). This remarkable variability in AFB_1_ biotransformation observed in these species can be explained with differences in the activity of hepatic enzymes involved in the biotransformation and detoxification processes considering both their expression and catalytic activity (Becker-Algeri et al., 2016). The average conversion value was 2.5% (Veldman et al., 1992) in high yielding dairy cows, which produced a daily amount of about 40 L of milk, were tested. Importantly, Veldman et al. (1992) found a direct relationship between the carry-over rate and the milk yield with a maximal 6.2% carryover rate. AFM_1_ is the most commonly detected aflatoxin in milk and the excretion of AFM_1_ depends on a range of factors including diet composition, rumen degradation and liver biotransformation capacities, the duration of lactation (Fink-Gremmels and van der Merwe, 2019) as well as on the animal breed and udder health status (Masoero et al., 2007). In dairy cows ingesting AFB_1_ contaminated feedstuffs, the excretoin of AFM_1_ occured in 12 – 24 h and up to 2 – 3 days in milk, whereas the AFM_1_ clearance in milk depended on several factors, mainly on the amount of ingested AFB_1_ and the duration of mycotoxin consumption with an excretion for a variable period of about 5 – 7 days from the ending of AFB_1_ assumption by cows (Masoero et al., 2007).

Well-reported variations in AFM_1_ contamination were observed in milk worldwide, which were dependent on several factors like geographical area, environmental and climatic conditions including seasons and weather, as well as on the diversity and level of development of farming systems and the consumption of feed concentrates and green forage (Becker-Algeri et al., 2016). In recent years and independently of the type of commodity, the occurrence of AFM_1_ in milk and dairy products was lower in Europe (for example in Italy, Portugal, Turkia, and Croatia) than in Asia or South America, where higher mycotoxin frequencies up to 100% were reported (Filazi and Sireli, 2013; Becker-Algeri et al., 2016). In Europe, low levels of AFM_1_ contamination were reported in milk, and only 0.06% of the analyzed samples were above the European limit of 0.05 μg/kg milk. Nevertheless, when such incidents occur a widespread AFM_1_ contamination of milk may develop, which has to be taken into account and adequately considered and controlled (van Asselt et al., 2017). In addition, risk managers should also consider that aflatoxin concentrations in milk may vary within the year and may also depend on the geographical location and climatic conditions. Finally, AFM_2_ has also been investigated in milk with different outcomes varying from its absence to a not negligible occurrence in powdered, UHT and pasteurized milk samples (Becker-Algeri et al., 2016).

The AFM_1_ contamination of dairy products is classified as an indirect contamination. For example, when the milk used in cheese-making was contaminated by aflatoxins, AFM_1_ unevenly distributes between whey and curd, because AFM_1_ prefers to bind to milk proteins, first of all to casein. For this reason, AFM_1_ is more concentrated in the curd and cheese than in the milk itself, which was used for cheese-making (Anfossi et al., 2012). Therefore, AFM_1_ levels were 3 – 8 times higher in certain dairy products than in the milk, and stable AFM_1_ residues were detected in the final dairy products like milk powder even after heat processing. In addition, the total amount of AFM_1_ does not change significantly during the cheese-making and cheese maturation processes but these steps influence the AFM_1_ and protein concentration ratios as a result of skimming and water loss (Anfossi et al., 2012). Although many studies on the contaminations of dairy products by AFM_1_ are available (Anfossi et al., 2012; Becker-Algeri et al., 2016) only few of them present any data estimating concentration factors for AFM_1_ in different cheeses. However, 2.5 – 3.3 and 3.9 – 5.8 times higher concentrations of AFM_1_ calculated on a weight basis were recorded in soft and hard cheeses, respectively, than those AFM_1_ concentrations found in the milk, from which these cheeses were made (Filazi and Sireli, 2013). In Europe, the food business operator has to justify and provide the specific concentration or dilution factors for AFM_1_ in the processed foodstuffs during official controls performed by the competent authority (EC Regulation, 1881/2016).

In this context, AFM_1_ contaminating milk should be unremitting to our attention and we should also take a special care of infants avoiding their exposures to AFM_1_ *via* milk and infant formulas (Fink-Gremmels and van der Merwe, 2019). Kerekes et al. (2016) emphasized the importance of regular control of produced milk and also the introduction of an appropriate action limit in combination with immediate corrective actions at the farm level. In fact, feed producers have to manage and control the feed ingredients intended for the production of feed for the lactating animals for risk mitigation. Feed ingredients should be selected based on their quality characteristics, whereas farmers, when the AFM_1_ content of milk exceeds the legal limit, have to withdraw milk consignments and also have to remove contaminated feedstuffs (Trevisani et al., 2014).

As far as the aflatoxin residues detected in edible tissues of bovine, pigs and poultry are concerned, these AFB_1_ entry routes do not contribute significantly to human aflatoxin exposures (Fink-Gremmels and van der Merwe, 2019). Nevertheless, data on the aflatoxin contents in the edible tissues of bovine species are scarce and it is generally assumed that aflatoxins are partly degraded in the rumen and they are rapidly metabolized in the liver after absorption from the intestines. The transfer rates of aflatoxins into the edible tissues of pigs are very low owing to the rapid pre-systemic and hepatic metabolisms, and the aflatoxin residues in pork are therefore not considered as of public health concern. Similarly, poultry with low levels of aflatoxin contaminations do not seem hazardous to humans although the presence of aflatoxin-residues in poultry liver is well-documented (Fink-Gremmels and van der Merwe, 2019). Importantly, a rapid decrease in AFB_1_ residues was observed in poultry muscles and liver after 3–7 days of uncontaminated dietary, significantly reducing the risk for human health (Filazi and Sireli, 2013). However, AFL is the main component of total AF residues in poultry with highest contents in liver (Frazzoli et al., 2017). In the case of laying hens, aflatoxins and their metabolites, particularly AFB_1_ itself and AFL, can also be carried over to eggs but very discrepant transmission ratios were reported in this case. Recent studies demonstrated very low amounts of aflatoxin residues in eggs, merely between 0.01% (Herzallah, 2013) and 0.07% (Hassan et al., 2012) of the aflatoxin intake. AFB_1_ residues appeared in eggs after 5 days of feeding with contaminated feedstuffs and the amount of AFB_1_ depended on the duration of feeding with contaminated grain. Similar to dairy products, the presence of aflatoxins in eggs may be indicative of the aflatoxins contamination of the feed.

## Aflatoxicoses and Animal Susceptibility

In general, mycotoxicosis refers to syndromes appearing after ingestion, skin contact or inhalation of toxic secondary metabolites produced by toxigenic molds belonging to the genera *Aspergillus*, *Fusarium*, and *Penicillium* as well as to some other fungal taxa (Gallo et al., 2015). Within mycotoxicoses, aflatoxicosis refers to any disease caused by the consumption of foods and feeds contaminated with aflatoxins. It is well-known that AFB_1_ is a potent mutagenic, carcinogenic, teratogenic, and immunosuppressive fungal secondary metabolite and all these effects may be linked to the interference of AFB_1_ and its derivatives with the synthesis of proteins, the inhibition of various metabolic pathways or to the onset of oxidative stress. All these disadvantageous physiological effects will lead consequently to damages in various organs, especially in the liver, kidney, and the heart.

Aflatoxicoses may emerge in any livestock but literature reports on outbreaks mostly in poultry, pigs, equine, sheep, and cattle. The exposure of domestic animals to AFB_1_ mainly occurs through the ingestion of contaminated feeds, however, skin contacts or inhalation exposures might also contribute (Gallo et al., 2015). It is well-known that ruminants are among the least susceptible animals to the negative effects of mycotoxins in comparison to monogastrics. However, the rumen has a saturable capacity of detoxifying aflatoxins by microflora, depending on (i) variations in the diet, (ii) the consequences of metabolic diseases, such as rumen acidosis, (iii) rumen barrier alterations as a result of animal diseases, and also (iv) the actual concentrations of aflatoxins present in the animal feed (Fink-Gremmels, 2008a). Consequently, clinical manifestations of aflatoxicoses in ruminants are associated typically with aflatoxins that are not degraded at all or not completely degraded by the rumen microflora.

Most of the data we have already had in our hands on mycotoxin toxicity are coming from experimental studies with purified compounds in otherwise healthy animals, which knowledge may help us with the early and reliable diagnosis of mycotoxicoses. However, when natural episodes of mycotoxicoses occur, versatile signs of disease could appear depending on the environmental conditions and also on several other features of the animals involved, including nutrition, sex and breed. For this reason, the diagnosis of mycotoxicoses is often difficult but it should rely on observing the clinical symptoms on the affected animals and also on analyzing the feed involved in the intoxication (Council for Agricultural Science and Technology [CAST], 2003). Given aflatoxins could act in synergy with other mycotoxins and also with other disease-provoking agents and, therefore, additional apparently unrelated pathological symptoms and even diseases are observed and reported in the affected animals. Furthermore, most mycotoxicoses including aflatoxicoses may present non-pathognomonic features and, consequently, there are no definitive diagnostic symptoms to orient farmers and veterinarians to assign aflatoxin exposures unequivocally to the death of animals. Obviously, even other otherwise unrelated diseases may trigger similar responses in the domestic animals to those of aflatoxins (Richard, 2008).

Aflatoxins do not affect all animals uniformly. Some animal species are inherently more resistant, such as sheep, goats and cattle, whereas other animals are more susceptible like swine, chickens, turkeys, and ducklings. In addition, considerable breed differences are documented within a given species (Richard, 2008), and the physiological responses to the adverse effects of aflatoxins are also influenced by age (young animals are usually more sensitive than elder ones and, in particular, piglets and chicks), sex, diet, and weight, exposure to infectious agents, and the presence of other mycotoxins or other pharmacologically active substances (Zain, 2011). In addition, when mycotoxins are present simultaneously, some interactive effects, classified as additive, antagonistic or synergistic, could also occur (Gallo et al., 2015).

### Animal Exposure to Aflatoxins

The exposure of animals to aflatoxins may trigger biological reactions that could be classified as acute, overt diseases with high morbidity and mortality, or, as it is usually the case, chronic, insidious disorders that impairs animal productivity (Bryden, 2012; Pierron et al., 2016). When livestock ingest aflatoxins the health effects could be acute, with severe consequences and evident signs of disease or even may be lethal when these toxins are abundantly consumed, even if this event is rare under farm conditions (Gallo et al., 2015). The timing of the proper diagnosis is a crucially important factor because the suspicious contaminated feed is likely consumed well before it can be tested (Council for Agricultural Science and Technology [CAST], 2003). The earliest clinical signs and lesions observed in turkey “X” disease, hepatitis “X” of dogs, and similar cases of acute aflatoxicoses were anorexia, lethargy, hemorrhages, hepatic necrosis, and bile duct proliferation (Miller and Wilson, 1994). Furthermore, the aflatoxins’ impact on animals should not be limited to the extreme effects of aflatoxicoses because it is related mainly to the chronic toxicity caused by the consumption of sublethal doses and to the fact that low levels of chronic exposures may result in cancer.

Considering the chronic effects of aflatoxins, hidden pathological alterations with reduced ingestion, productivity and fertility were implied, including lowered milk, meat, and egg productions, decreased weight gains and/or unclear changes in animal growth, feed intake reductions or feed refusals, alterations in nutrient absorption and metabolism, various typologies of damages to vital body organs, disadvantageous effects on the reproduction and endocrine systems and also suppression of the immune system with subsequently increased disease incidence. The economic consequences of chronic aflatoxicoses are many times larger than those of the rare acute cases with immediate morbidity and lethality (Council for Agricultural Science and Technology [CAST], 2003).

### Hepatotoxic, Carcinogenic and Mutagenic Effects

Among the major devastating effects of aflatoxins on animals, these harmful metabolites specifically target the liver and, hence, are proved to be primarily hepatotoxic. In acute aflatoxicosis, the emerging clinical symptoms of acute hepatic injury include coagulopathy, increased capillary fragility, hemorrhage and prolonged clotting times. Gross liver changes are caused by hemorrhage, centrilobular congestion, and fatty changes in surviving hepatocytes. Death of the poisoned animal may occur within hours or a few days after exposure. In broiler chicks, hemorrhagic anemia syndrome develops as characterized by massive hemorrhagic lesions in major organs and musculature even if the anemia could be considered as a secondary effect of severe hypoproteinemia caused by primary liver damage (Council for Agricultural Science and Technology [CAST], 2003). However, changes in extrinsic coagulation factors as determined by increased fibrinogen concentration were also reported in lambs (Zain, 2011). In addition, in broiler chicks, other reported clinical signs of aflatoxicosis were glomerular hypertrophy, hydropic degeneration of tubuler epithelium in kidneys and increases in the number of mesengial cells, as well as atrophy and lymphoid depletion in the thymus and bursa of Fabricius (Ortatatli and Oguz, 2001).

Even in chronic aflatoxicosis, most of the effects can be attributed to hepatic injury but with milder symptomes and icterus can also be observed. The pathological alterations in the liver mostly consist of degenerative changes and circulatory disturbances and also include a yellow to brassy color, enlarged gall bladder, diluted bile, histological signs of fatty changes in the hepatocytes, bile duct proliferation and periportal necrosis. In chronic aflatoxicosis, the signs are so protean that the episode may go undiagnosed for long periods of time (Pier, 1992). Because aflatoxins metabolized in the liver, the histological changes are observed primarily within this organ. Not surprisingly, centrilobular hepatic necrosis or hepatocellular vacuolar change and bile duct proliferation are consistent lesions in cow, sheep, goat and swine. Hepatic fibrosis has been reported in all species when the animals did not die from acute aflatoxicosis (Miller and Wilson, 1994). In Piedmontese calves, an outbreak of hepatic encephalopathy consequent to aflatoxin intoxication is to be mentioned: neurological signs varying from comatose or depressed mental status, spinal hyporeflexia, wasting and proprioceptive deficits, and compulsive behavior characterized by anteropulsion and right circling in large circles (D’Angelo et al., 2007).

Aflatoxins are also carcinogenic in animals and aflatoxin B_1_ is the most powerful liver carcinogen known for rats. AFB_1_ and AFG_1_ possess an unsaturated bond at the 8,9 position on the terminal furan ring (Figures 1, 4), and epoxidation at this position results in a reactive species, which induces oxidative stress of tissues, depletes antioxidants, forms DNA adducts and, hence, initiates malignant transformations. AFB_2_ and AFG_2_ are relatively less toxic unless they are metabolically oxidized first to AFB_1_ and AFG_1_ *in vivo*. Chronic exposure to low doses of aflatoxins is one of the major risk factors in the etiology of hepatocellular carcinoma, and all animal models exposed to AFB_1_ have developed this type of cancerous desease thus far. Aflatoxins have been reported to cause other malignancies as well, including adenomas of esophagus, trachea, kidney and lungs, carcinoma of the pancreas and osteogenic sarcomas (Yilmaz et al., 2018). However, the carcinogenicity in farmed animals cannot be detected because of the relatively short period of time, in which the animals are fed prior to marketing (Richard, 2008). In addition, the chronic form of aflatoxicosis includes teratogenic effects in animals, which are associated with congenital malformations and, in the fetuses, multiple skeletal anomalies as incomplete ossification of skull bones and failure of ossification of long and flat bones, as well as delay in the intramembranous ossification process, defects in the vertebrae formation or their reduction in size. Other mutagenic effects of aflatoxins cover mutations in genes, alterations of DNA by chromosomal breaks, rearrangement of chromosome pieces or even acquisition or loss of entire chromosomes (Fetaih et al., 2014).

### Immunotoxic Effects

Although aflatoxins are primarily known as hepatotoxins and hepatocarcinogens, they have notable immunotoxic effects as well making animals more susceptible to many bacterial, viral, fungal and parasitic infections, as well as to the reactivation of chronic infections or reductions in vaccine and therapeutic efficacies (Oswald et al., 2005). Poultry (chickens and turkeys), pigs and in particular lambs are susceptible to induced immunosuppression due to aflatoxin exposure. Aflatoxins could impair both the cellular and humoral immune systems. *In vitro* and *in vivo* studies have demonstrated that AFB_1_ is immunotoxic, exerting its action particularly on cell-mediated immunity through (i) reducing the number of circulating lymphocytes, (ii) the inhibition or suppression of lymphocyte blastogenesis, (iii) impairing both cutaneous delayed-type hypersensitivity and graft versus host reaction and (iv) the modification of the activities of natural killer cells and of macrophage functions through the inhibition of phagocytosis, the expression and secretion of cytokines (TNF-α, IL-1β, IL-6, IL-10, and IFN-γ), and also by reducing intracellular killing as well as the spontaneous production of oxidative radicals (Council for Agricultural Science and Technology [CAST], 2003; Oswald et al., 2005; Meissonnier et al., 2008). The general mechanism of the immunosuppressive effects of AFB_1_ appears to be directly associated with the impairment of the synthesis of proteins. In fact, AFB_1_ is transformed *in vivo* into metabolites, which are able to bind actively DNA and RNA, to impair the activity of DNA-dependent RNA polymerase and also to inhibit the synthesis of both RNA and proteins. These inhibitory mechanisms have direct and indirect effects on the proliferation and differentiation of the lymphoid system cells and on the synthesis of cytokines involved in the regulation of the immune system (Oswald et al., 2005). An alteration of the inflammatory responses with a reduced synthesis of pro-inflammatory cytokines and an increase of anti-inflammatory cytokines was reported in weanling piglets fed for 4 weeks with low doses of aflatoxin (Marin et al., 2002). The effects of aflatoxins on humoral immunity are not so clear as their effects on cell-mediated immunity, and these differences are hardly recognizable between the different animal species unless higher doses of aflatoxins were introduced (Council for Agricultural Science and Technology [CAST], 2003; Meissonnier et al., 2008). Suppression of humoral immunity has also been recorded after observing decreases in lymphocyte infiltration, hemagglutination and in serum protein levels (Rushing and Selim, 2019). In pig, no major effects on humoral immunity were observed after AFB_1_ exposure but delayed and decreased ovalbumin-specific proliferation, suggesting an impaired lymphocyte activation (Pierron et al., 2016). However, a biphasic effect of AFB_1_ was shown in piglets and broiler chicks, with immunosuppressive effects observable during acute exposures and with inflammatory response with stimulatory effects depending on the doses, more precisely, low doses of AFB_1_ caused immunosuppression meanwhile high doses of it stimulated the immune system (Marin et al., 2002; Yunus et al., 2011). In details, piglets showed decreased leukocyte counts when exposed to low AFB_1_, and an increase in leukocytes with a high dose (Marin et al., 2002). This immunotoxic effect has significantly disadvantageous consequences on the health of farmed animals, via increasing both the susceptibility and the severity of infections like coccidiosis, salmonellosis and *Cryptosporidium bailey* infections in chicken, *Erysipelothrix rhusiopathiae*, *Brachyspira hyodysentariae*, and *Escherichia coli* infections in pigs, the reactivation of chronic infection by *Toxoplasma*, and the impairment of vaccination efficacies for *Bordetella bronchiseptica* and *E. rhusiopathiae* or with the model antigen ovalbumin in swine (Oswald et al., 2005; Pierron et al., 2016), as well as for fowl cholera and Mareck disease in chickens and/or turkeys (Oswald et al., 2005).

### Nephrotoxic Effect

Renal damages have also been reported after long-term administration of aflatoxins with the symptoms of inflammation, cell necrosis, and toxicosis, which may increase the weight of kidneys and may induce congestion in renal sinusoids. The kidneys are one of the target organs of aflatoxins, and their toxicity is activated by oxidative stress that alters the expression of proline dehydrogenase reducing the proline levels, which induces downstream apoptotic cell death. Moderate focal to diffuse necrosis in the renal tubules and increased renal tubular cells, which may be filled with bile pigments, hyaline, and lipid, with occlusions of their lumens with local edematous changes were reported in the kidneys of aflatoxin-exposed rats (Li et al., 2018). In poultry, the toxic effects exerted by AFB_1_ on renal functions included reduced concentrations of calcium, inorganic phosphate, sodium, and potassium and increased levels of urea, creatinine and uric acid (Yilmaz et al., 2018). In addition, AFB_1_ was reported to cause severe heart damage with tachycardia, tachypnea and even death, although the exact mechanism of cardiotoxicity has not been completely known.

### Reproductive Effects

Not so long time ago, the harmful effects of aflatoxins on animals did not include any direct impairment of reproduction but indirect effects through other physiological systems have been considered. Nevertheless, more recent animal studies suggested that aflatoxins should also induce direct reproductive toxicity in both male and female animals based on adverse effects to both spermatozoa and oocytes. Following aflatoxin exposure *in utero*, monitoring growth parameters in baby animals indicated growth retardation, reduced fetal or egg weights and reduced fetal lengths of the offspring animals. In piglets exposed to maternal aflatoxicosis, growth retardation, thymic involution and impaired peripheral immune efficiency were events frequently reported and leading to early death (Mocchegiani et al., 1998), whereas broiler hens exposed to aflatoxin resulted in embryonic mortality and lowered the immunity in the progeny chicks (Rawal et al., 2010). In addition, aflatoxins also possess spermatotoxic effects, which have an impact on the morphology and physiology of spermatozoa: AFB_1_ affects the male reproduction system altering spermatogenesis as well as epididymal and Leydig cell functions, and also reducing the production of testosterone and the fertility in rats, birds and cattle (Agnes and Akbarsha, 2003). In females, AFB_1_ reduces the fertility of oocytes by the disruption of oocyte maturation through epigenetic modifications as well as oxidative stress, excessive autophagy and apoptosis (Liu et al., 2015). In addition, in poultry, worsening egg production and quality, together with the deposition of aflatoxins residues in the eggs are described in both acute and chronic aflatoxicoses. The lowered egg production was attributed to the aflatoxins’ effect on liver metabolism and function as well as liver lesions in layers, to the inhibited synthesis of proteins and lipogenesis, and to decreased feed intake and digestibility (Jia et al., 2016). It is well-known that aflatoxin causes alterations in the carbohydrate metabolism and impairments of the lipid transport, which effects result in decreased glucose levels and reduced lipid accumulations within hepatocytes, as well as pathological alterations in serum biochemistry and of most coagulation factors have been described in poultry, pigs, cattle and rabbits.

### Gastrointestinal Dysfunctions

Aflatoxins modulate and affect the GIT in multiple ways, the most important of which are changes in the gut morphology, the digestive ability or activity of digestive enzymes, intestinal innate immunity and gut microbiota (Mughal et al., 2017). Nevertheless, only few reports are available in this field and the presented data are also controversial in many cases, especially for ruminants. The absorption of aflatoxins across the intestinal barrier is maximal in the upper part of the GIT in non-ruminant animals whereas in ruminants, the harmful aflatoxins like AFB_1_ are transformed to less toxic compounds (e.g., AFM_1_) or to metabolites with similar or even higher toxicity than the parent molecules (e.g., aflatoxicol) (Gallo et al., 2015). Among the overall adverse effects of aflatoxins, the most significant ones are related to the growth of animals and result in reduced performance. Aflatoxins cause reduced feed intake or even feed refusal with a subsequent decrease in body weight gain, which is determined by direct and/or indirect effects of aflatoxins on the nutrient quality, digestibility and/or absorption. During AFB_1_ exposure, piglets showed reduced weight gain and Japanese quail have shown a reduction in egg weight (Marin et al., 2002). Reduced absorption of nutrients was reported after aflatoxin exposure and, in cattle, this decreased feed efficiency contributed to the observed compromised ruminal function by reducing cellulose digestion, volatile fatty acid production and rumen motility (Zain, 2011). In relation to nutrient digestibility and metabolizable energy, the presence of aflatoxin in dietary was suggested to reduce the apparent digestibility of crude proteins in ducks, to increase amino acid requirements and to reduce energy utilization in terms of net protein utilization and apparent digestible and metabolizable energy in ducks and chickens. Aflatoxins modulate the activity of digestive enzymes but contradictory effects were reported for amylase, trypsin and chymotrypsin activities in pancreas and duodenum with unchanged level of nutrient digestion in the intestine. However, aflatoxins seem to have only moderate affects on or even sometimes do not affect at all the growths of animals through the alteration or modulation of digestive functions (Grenier and Applegate, 2013), even if, in broiler chicks feed with experimental AFB_1_ diet, impaired growth, major serum biochemistry measures, gut barrier, endogenous loss, and energy and amino acid digestibility were reported (Chen X. et al., 2016). The effects of AFB_1_ on intestinal epithelium and microbiota were investigated in some *in vivo* studies in broiler chicken and rodents. The density of the whole intestine was reduced in the case of low AFB_1_ doses but at higher doses no such changes were recorded, instead the number of apoptotic cells in the jejunum were elevated, jejunal villi presented lower height, and intestinal lesions were observed in duodenum and ileum, with leucocytic and lymphocytic infiltration. Meanwhile, reduced microbial diversity was observed in the colon with adverse effects on lactic acid bacteria *versus* unchanged proportion of *Firmicutes* and *Bacterioidetes* (Robert et al., 2017).

Additional symptoms of aflatoxicosis involved malnutrition. *In vitro* methods and animal models, predominantly, in piglets and broiler chicks, have showed that AFB_1_ altered bioavailability and distributions of essential metal ions as zinc, calcium, magnesium and potassium, reduced the activities of lipogenic and amino acid metabolizing enzymes leading to reduced lipogenesis, and reduced serum concentrations of 25-hydroxy vitamin D, 1,25-dihydroxy vitamin D and calcium, consequently altering renal functions and parathyroid metabolism (Rushing and Selim, 2019).

Finally, aflatoxicosis in horses showed non-specific clinical signs, such as inappetence, depression, fever, tremor, ataxia and cough. Meanwhile, at necropsy, yellow-brown liver with centrilobular necrosis, icterus, hemorrhage, tracheal exudates and brown urine were observed (Caloni and Cortinovis, 2011).

## Qualitative and Quantitative Aflatoxin Analytical Methods – Economic Significance of Analysis

Since the massive death of turkeys (Turkey-X deceases) recorded in England in 1960, a wide spectrum of research has been launched and carried out to shed light on the causes of such high mortality (Büchi and Rae, 1969; Rodricks and Stoloff, 1977). Deciphering the factors leading to Turkey-X disease is a fascinating illustration of how a multidisciplinary approach may help us to solve an important animal health problem. The research covered the development of new analytical tools to measure mycotoxins more precisely, the exploration of the physiological and toxicological effects of these harmful compounds as well as the efficient removal of the toxins and setting up to prevent the manifestation of and to cure the disease itself (Forgacs and Carli, 1962).

Mycotoxins are mainly produced on small grains, cereals such as wheat, barley, oats, rye and triticale or on corn but animal products such as milk, meat, liver or eggs can also be contaminated by mycotoxins at various points of the feed and food chain (Gacem and El Hadj-Khelil, 2016; Udovicki et al., 2018). Because the sampling of feeds and foods for mycotoxin analysis may follow quite different protocols in different laboratories the standardization of these procedures represents a real challenge for analytics. During mycotoxin analysis, extraction and detection are crucially important issues to gain reliable analytical data, which may help us to optimize storage conditions and setting up rules to control mycotoxin production (Yao et al., 2015).

The first step in the analysis is to extract mycotoxins from the sample after correct sampling and sample preparation. The former and traditional extraction methods for aflatoxin analysis gave us a sample matrix in which the HPLC analysis was too complicated to carry out because of the presence of disturbing and interfering components (Kamimura et al., 1985). Later, the clean-up immonoaffinity columns containing gel suspension of monoclonal antibodies gained ground and became popular due its high specificity. The suspension retains the aflatoxin molecules what can be eluted cleanly, free from any disturbing compounds (Borbély et al., 2010; Lai et al., 2014). Another intention is the extraction with different solvents such as carbon tetrachloride (CCl_4_), chlorobenzene (C_6_H_5_Cl), chloroform (CHCl_3_), and dichloromethane (CH_2_Cl_2_), methanol and acetonitrile (Sepherd, 2009; Bertuzzi et al., 2012; Sarnoski et al., 2015).

Analytical methods of mycotoxin surveillance are wide-ranging and may vary within broad limits across countries. As a result of a community effort having been made to unify surveillance regulations in the European Union, the European Commission (2006) Regulation (EC) No. 401/2006 laid down the requirements for both recovery and precision in different toxin concentration ranges and gives the methodology for the validation of any analytical procedure, making possible to check if it is acceptable for official analysis (EC No. 401/2006). This covers all characteristics required for an analytical method with such a specific sample background, and the list of characteristics ranges from accuracy to measurement uncertainty through the limit of detection (EC No. 401/2006; Sheppard, 2008; Alshannaq and Yae-Hiuk, 2017; Shanakhat et al., 2018).

An overview on the available analytical methods can be given based on the remarkably abundant literature having been published in this field. We have a plethora of quantitative methods ranging from the different types of Thin Layer Chromatograpy-based to different varieties of HPLC to LC-MS/MS-based methodologies. In addition, we can also find good performance procedures among semi-quantitative methods like ELISA-based or biosensor-based protocols. Emerging technologies include hyperspectral imaging and aptamer-based biosensors (EC No. 401/2006; Sheppard, 2008; Vidal et al., 2013; Alshannaq and Yae-Hiuk, 2017; Shanakhat et al., 2018).

The performance parameters of different aflatoxin analytical methods are summarized in Table 5. The different methods can be characterized by several parameters such as accuracy, applicability, reproducibility, limit of detection and so on (Sheppard, 2008; Alshannaq and Yae-Hiuk, 2017; Shanakhat et al., 2018). Trucksess and Zhang (2016) argued that all practically useful analytical methods should meet the basic guidelines of reproducibility in different laboratory settings. Based on these premises, protocols that are used in different laboratories from sampling to analysis were compiled, and systems relying on certified material samples (CRMs) are also closely related to this.

**TABLE 5:** Analytical methods for aflatoxin measurement.

**Type of method**	**Technique**	**LOD**	**References**
Quantitative methods	Thin Layer Chromatography combined with scanner	0.1 μg/kg B_2_; G_2_; M_1_, 0.2 μ/kg B_1_; G_1_;	Kamimura et al., 1985
	High Performance Liquid Chromatography, in combination with fluorescence detector	0.002 μg/kg	Kilicel et al., 2017
	Liquid Chromatography with Mass Spectrometry	0.5 μg/kg	Sirhan et al., 2013
	Liquid Chromatography with tandem Mass Spectrometry	1 μg/kg	Alsharif et al., 2019; Ouakhssase et al., 2019
	Ultra High Performance Liquid Chromatography with fluorescence detector	0.02 μg/kg	Beltrán et al., 2011; Cui et al., 2017
	Capillary electrophoresis	1 μg/kg 0.1 ng/g	Arroyo-Manzanares et al., 2010; Xiao et al., 2018
Semi-quantitative methods	ELISA	1 ng/l	Huybrechts, 2011
	Lateral flow tests LFT	5 μg/kg	Goh et al., 2014,
	Direct fluorescence	5 μ/kg	Wacoo et al., 2014
	Fluorescence polarization immunoassay	30 ng/ml	Maragos, 2009
	Biosensors	0.05 ml 0.005 μg/l	Gurban et al., 2017; Man et al., 2017
Indirect methods	Spectroscopy	4 μg/kg	Wacoo et al., 2014
Emerging technologies	Hyperspectral imaging	10 μg/kg	Wang et al., 2014
	Electronic nose	5 μ/kg	Ottoboni et al., 2018
	Aptamer-based biosensors ECL	0.1 pg/ml	Shim et al., 2014; Castillo et al., 2015; Guo et al., 2016; Jia et al., 2019; Kordasht et al., 2019;

Currently, a number of HPLC-MS or MS/MS equipment are used world-wide to gain a detailed overview on the mycotoxin spectra in feeds and foods depending on laboratory capabilities (Berthiller et al., 2018). At the same time, ELISA methods and equipment are used for quick mycotoxin measurements (Christoforidou et al., 2015; Xu et al., 2015; Sineque et al., 2017). New developments in this field have been published in the latest literature (Pennington, 2017; Udomkun et al., 2017; Yan et al., 2017). For example, a novel and promising method has been presented to detect aflatoxin B_1_, B_2_ and ochratoxin A in rice starting with dispersive liquid–liquid microextraction followed by LC and fluorescence detection (Lai et al., 2014; Adi and Matcha, 2018).

The impact of aflatoxins on human health (Theumer et al., 2018; Omotayo et al., 2019) is far the most important challenge, which we should keep an eye on in the whole feed and food chain (Zheng et al., 2018). This is the reason for why aflatoxin-related research including analytics is flourishing today. Future research should aim at a deeper understanding of the high-complexity and multi-parameter processes influencing the aflatoxin contents of feeds and foods. Novel multilateral approaches are definitely needed to control mycotoxins and their disadvantageous agricultural, health care and economic impacts more effectively (Krska et al., 2016; Stadler et al., 2018).

## Author Contributions

IP and ZG acquired funding, managed methodology, contributed to writing, reviewing and editing of the manuscript. FP, PS, WP, GP, TG, FG, AS, and ZG contributed to draft preparation, writing, editing and visualization. IP, PS, WP, and FP finalized the manuscript.

## Conflict of Interest

The authors declare that the research was conducted in the absence of any commercial or financial relationships that could be construed as a potential conflict of interest.
